# Analyzing the etiology behind mortality associated with antepartum, intrapartum, and post-partum cases in a tertiary care teaching hospital of West Bengal

**DOI:** 10.4274/jtgga.2017.0136

**Published:** 2018-06-04

**Authors:** Md Illias Kanchan Sk, Aparajita Chattopadhyay, Ankit Anand, Tapan Kumar Naskar, Somajita Chakraborty

**Affiliations:** 1 Department of Population Policies and Programmes, International Institute for Population Sciences, Mumbai, India; 2 Department of Development Studies, International Institute for Population Sciences, Mumbai, India; 3 Institute for Social and Economic Change, Bangalore, India; 4 Department of Obstetrics and Gynecology, Medical College and Hospital, Kolkata, India

**Keywords:** Maternal death, facility-based, West Bengal, eclampsia

## Abstract

**Objective:**

The study was undertaken to understand the causes and circumstances of maternal deaths in West Bengal.

**Material and Methods:**

One hundred ten maternal deaths were reported during the period December 2010 through June 2012 in the Maternity Ward of Medical College and Hospitals, West Bengal. These deaths were reviewed using a facility-based Maternal Death Review protocol. The number and percentages were calculated and binary logistic regression analysis was performed.

**Results:**

The majority of the deaths occurred in the 20-24 years’ age group, those with Hindu religion, in the first and second gravida, and the postpartum period. One third of mothers had cesarean sections. The majority (78.2%) of deaths were among referred cases. Eclampsia was the leading cause of maternal death (29.1%). Approximately half of the deceased women sought care after 10 hours of developing complications. More than one-third of maternal deaths were registered with type 1 delays.

**Conclusion:**

Our study demonstrates that maternal deaths occurred among young women, referred cases, with cesarean sections and type 1 delays. We recommend that imparting basic skills and improving awareness to the community about the danger signs of pregnancy could be an effective measure to detect maternal complications at an earlier stage.

## Introduction

Everyday 830 women die due to pregnancy- or childbirth-related complications around the globe (1). According to a World Health Organization report, approximately 303,000 women died during pregnancy or its related complications in 2015. Developing countries accounted for approximately 99 percent of the global burden of maternal deaths (2). Every year, India contributes around 45,000 maternal deaths, which is the second largest number of maternal deaths after Nigeria (2). Maternal mortality ratio (MMR) is defined as ''the number of maternal deaths per 100,000 live births'' (2). In 2015, the MMR in India was estimated as 174 (2).

According to the latest Registrar General India-Sample Registration System survey report in 2013, the MMR in West Bengal was 117 (3). West Bengal (19.86 percent) has the lowest percentage fall in MMR compared with the other states of India (4). Many deaths still occur in West Bengal due to eclampsia, hemorrhage, severe anemia, obstructed labor, and puerperal sepsis (5,6,7). Thus, pregnancy-related complications continue to have an enormous effect on the life of mothers and their infants.

To achieve the development goal pertaining to maternal and child health requires an increase to access and the coverage of key interventions and improvements in the quality of care (QoC) (8). Maternal death reviews in health facilities, which is also called maternal death audit, helps to understand the problem of the importance of QoC. This type of audit identifies obstetric causes of maternal mortality and provides detailed information on avoidable factors related with maternal deaths (9). Analysis of these deaths by using the facility-based maternal death review (FBMDR) approach also gives a clear picture of the different types of delay that lead to deaths among pregnant women at different stages (10,11). The maternal death review helps to understand the complex reasons of these women's death and to set actions to address problems for improving QoC, and ultimately, to save lives in the future.

This facility-based study was performed to understand the causes and circumstances of maternal deaths in a tertiary care hospital of West Bengal so that corrective measures to reduce preventable maternal deaths could be suggested in that health setup. 

## Material and Methods

### Study settings

The selected teaching hospital is Asia's oldest medical college, situated in eastern part of India, which serves as a major tertiary care center in West Bengal. This medical college serves the facilities of antenatal care and inpatient medical care for pregnant women. The doctors and the other health workers are qualified and experienced in handling maternal cases. Patients mostly come to this medical college and hospital in critical condition and in a moribund state from other health care units. Therefore, the hospital can give a representative sample to understand the cause and determinants of maternal death in the community as a whole. 

### Materials

The present study sought to understand the etiology of maternal deaths in West Bengal using the FBMDR approach. All 110 deaths that occurred between December 2010 and June 2012 in the Maternity Ward of the Medical College and Hospital, West Bengal, India, were analyzed. Deaths among reproductive-aged women (15-49 years) due to pregnancy-related complications and childbirth and occurring within 42 days of delivery were considered as maternal deaths. The maternal death review form and bed head tickets were used to acquire the information about the deceased women. We do not routinely follow thromboprophylaxis in our hospital because most pregnant mothers present as underweight at the time of admission.

### Statistical analysis

The number and percentages were calculated to understand the causes and characteristics of maternal deaths. Logistic regression was performed to calculate the odds ratios of being unstable at the time of admission, experiencing any delay, Delay 1 (seeking care) and Delay 2 (reaching first level health facility). We considered age, gravidity, religion and referral status of the patient as independent variables. The level of significance was taken as p<0.1, p<0.05, and p<0.01. Microsoft Excel 2007 and IBM SPSS version 20 were used to analyze the data. 

### Ethics committee approval

Permission and ethical clearance for using the data were obtained from the ethics review committee of the selected Medical College and Hospital. No personal information of the patients was used. The data were analyzed anonymously. 

### Informed consent

We used the secondary data from the hospital. We did not interview/communicate with any patient. The ethics review committee waived the need for informed consent. 

## Results

The study found that the eclampsia accounted 29.1%, which made it the leading cause of maternal mortality, followed by hemorrhage (22.7%) and infections/sepsis (10.9%) (Table 1). About 80% of women died of direct causes. Among the indirect causes (16.4%), jaundice (8.2%) and anemia (3.6%) were the major conditions. There were four women out of 110 women whose causes of death had not been identified.

Out of 110 maternal deaths, 60% were from the Hindu religion and the remaining 40% from the Muslim religion (Table 2). The number (about 46%) of women in the 20-24 years’ age group was higher compared with the other age groups. More than one-third of maternal deaths were second gravida. The majority of maternal deaths were reported in primiparas women (42.7%), followed by nulliparous women (34.5%). Out of 110 maternal deaths, twenty women had at least one abortion in the past and five women had two abortions. Nearly half of the women had no living children. The majority of deaths (78.4%) were among referral cases. Among the referrals, most came from subdivision hospitals/community health centers or rural hospitals. The study found that about half of the deceased mothers had received at least one antenatal examination.

Nearly half of the deceased women came to the medical college at the intrapartum stage (Table 3). Seventy-seven women died during the post-partum period and 11 died during labor (intrapartum). Pre-eclamptic toxemia and eclampsia (29.1%) was the main reason for admission, whereas 19.1 women had to come due to medical conditions. Half of the women (n=55) were unconscious at the time of admission and 38 women (34.5%) had cesarean sections. However, one-third of patients delivered vaginally without any assistance; 18 women died before they had delivered. 

**Figure 1:**
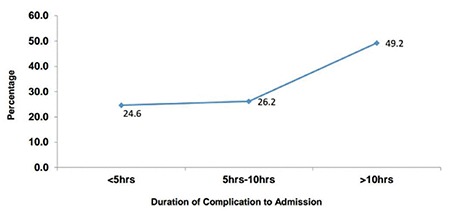
Percent distribution of women according to the duration of complication of admission (n=110)

**Figure 2:**
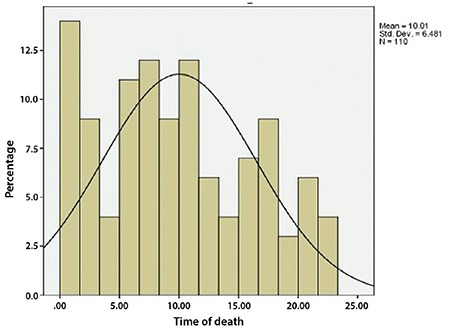
Percent distribution of women according to the timing of death (n=110)

About half of the women sought care after 10 hours of developing complications (Figure 1). The percentages of deceased women who sought care within the first five hours and after five to 10 hours were 24.6% and 26.2%, respectively. About 50 percent of the maternal deaths occurred between 12:00 am and 10:00 am (Figure 2).

Among all maternal deaths, 37, 28, and 13 had Delay 1 (delay in deciding to seek care), Delay 2 (delay in reaching first level health care facility) and Delay 3 (delay in receiving adequate care in facility), respectively (Table 4). In most of the cases of maternal deaths, multiple types of delay were co-existing. Delay in decision-making, illiteracy, and ignorance were the major contributors of first level delay. Both Delay 1 and Delay 2 were reported in 15 cases, and 5 cases had all types of delays.

The non-referred cases were less likely to be unstable at the time of admission compared with refereed cases (Table 5). In addition, any kind of delay increases the likelihood of being unstable at the time of admission by 2.6 times. It was observed that as the age of women increases the likelihood for any delay and the first delay decreases (Table 6). As compared to Hindu, Muslims were two times more likely to have the second delay.

## Discussion

Currently, our understanding of maternal death and its associated factors is very poor, partly due to the scarcity of data related to maternal deaths and its determinants (12,13). Looking at the enormity of the issue, we tried to explore the relationship between maternal death and associated factors. This attempt was made using the facility-based maternal death review approach for determining the causes of maternal deaths and its circumstances in West Bengal. 

The most common cause of maternal death was hypertensive disorders of pregnancy or eclampsia (29.1%), followed by hemorrhage (22.7%). Most of the studies conducted in West Bengal found that the eclampsia was the leading cause of maternal death in West Bengal (5,6,7,14,15,16). It is also known that a large number of maternal deaths at the time of pregnancy and childbirth occur due to hemorrhage in India and the world (17,18,19,20,21,22,23,24). 

The present study revealed that about 46% of the maternal deaths were recorded among women between 20 and 24 years. Out of all deaths, 60% of mothers were from the Hindu community and the remaining 40% were Muslims. Several studies reported that higher numbers of maternal deaths were found in the 20-24 years’ age group (6,17,24,25,26) among the Hindu community (17,26). The majority of maternal deaths (more than one-third) were second gravida and 13.6% women were fourth or higher gravida (27). More maternal deaths were reported in primiparous women (42.7%) compared with multiparous (about one-quarter) (27). This is comparable to the findings of other researchers who reported the highest proportion of maternal deaths occurred among multiparous and multigravida women (6,26,28).

An overwhelming majority of the deaths (78.2%) were among referral cases; most of such referrals were from subdivision hospitals/rural hospitals or community health centers and were in critical or irreversible condition at the time of admission. More than two-thirds of the women (70%) died following delivery and approximately half of the deceased women had sought care more than 10 hours after developing complications. Other studies also found high numbers of deaths among referred cases and within the first 24 hours of hospitalization (6,24,25,27,28,29). Similar to our findings, other studies have also shown that the majority of maternal deaths occur in the post-partum period (16,30). 

Fifty-five percent of women had attended at least one antenatal care examination; 19% had not undergone any examinations. The majority of women had reported that lack of awareness was the major cause for not receiving antenatal care. In a study, it was found that the majority of women (70%) had not received antenatal care during their pregnancy period (27). The study found more than one third of maternal deaths occurred with cesarean section (6,7,31).

This study also reveals that delays at various stages led to deaths. Type 1 delay was the major contributor in maternal deaths, about one-third of the women died due to this, followed by type 2 and type 3 delays. In a study, it was reported that type 1 and 2 delays had each influenced 58% of maternal deaths, and type 3 delays were registered in 46% of deaths (25). Comparable findings have been reported in a study where type 3 delays were the major contributor of maternal deaths (32). As compared with Hindus, Muslims were two times more likely to have type 2 delays, which was the delay in reaching the health facility. With the increasing age of the mother, the chance of delay decreases. Most of the deaths occurred at midnight and in the early morning, which is a matter of concern. 

The most important question is why do women die even after reaching the hospital? The fact that women die in hospital raises important issues of delays in referral to the Medical College and Hospital. These issues also pose a question about the availability, competence, and skills of the medical staff, as well as their attitude towards people at the level of referral. It should be understood why Muslim women face more delays in reaching the health facility. Why is eclampsia the leading cause of death? Another question can be raised: why did most of the maternal deaths occur at midnight and in the early morning? This issue compels us to think about the availability of medical staff and doctors at the referral level.

There is a need to understand the reason behind higher second type of delay among Muslim women. Better understating of causes of maternal deaths remains incomplete without prior knowledge of non-medical factors. It is a well-established fact that facility-based maternal mortality statics fail to reflect the true extent of mortality because medical causes are mostly determined, correlated, and predefined by social, cultural, demographic and health-seeking behavior of the population.

Our study demonstrates that maternal deaths occurred among young women, referred cases, with cesarean sections and Type 1 delays. We recommend that imparting basic skills and improving awareness to the community about the danger signs of pregnancy could be an effective measure to detect maternal complications at an earlier stage. Further, health infrastructure improvement attention should be paid to Muslim areas to eliminate the second type of delay.

## Figures and Tables

**Table 1:**
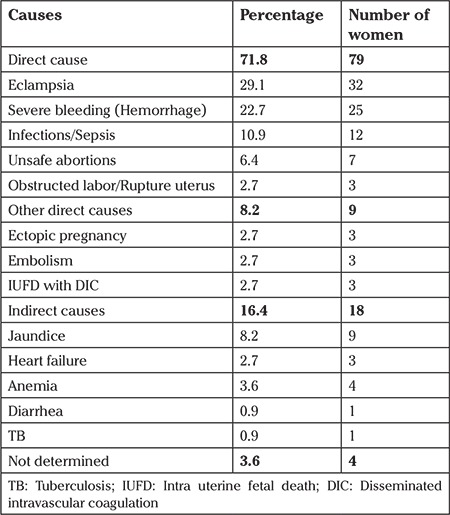
Distribution of causes of maternal deaths (n=110)

**Table 2:**
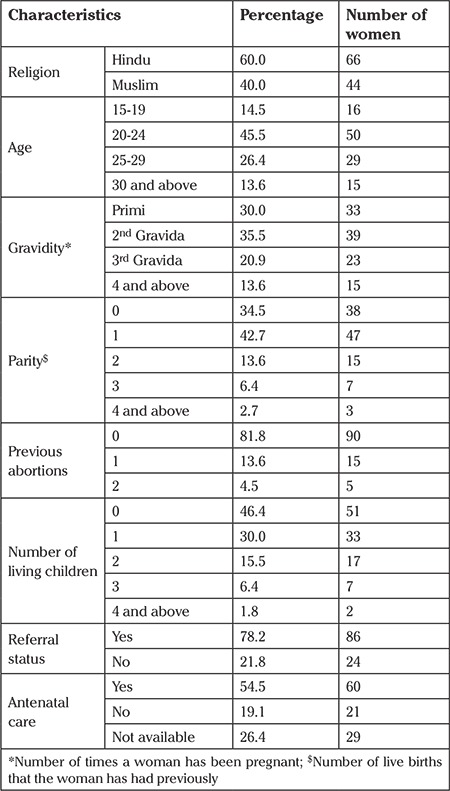
Sociodemographic characteristics and health-seeking behavior of the deceased women (n=110)

**Table 3:**
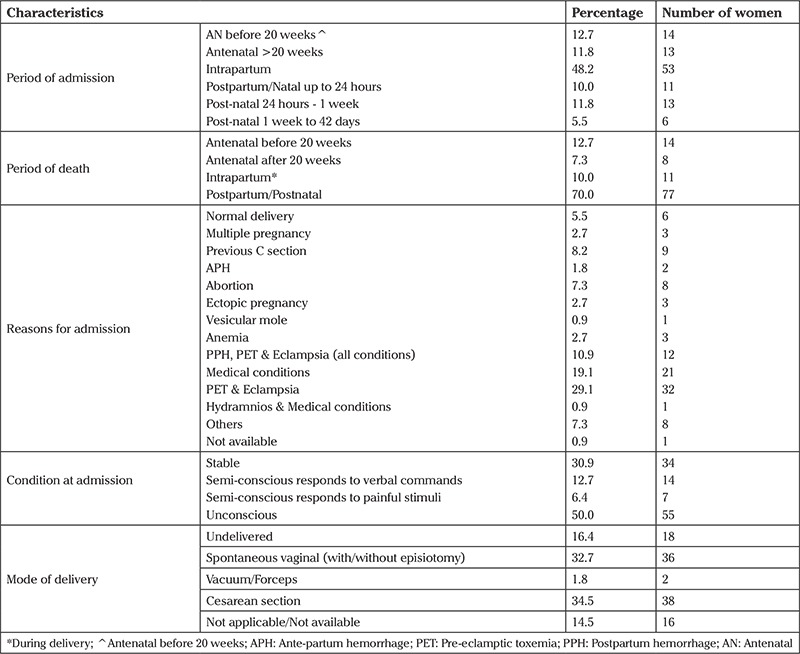
Obstetric complications, labor, and delivery status of the deceased women (n=110)

**Table 4:**
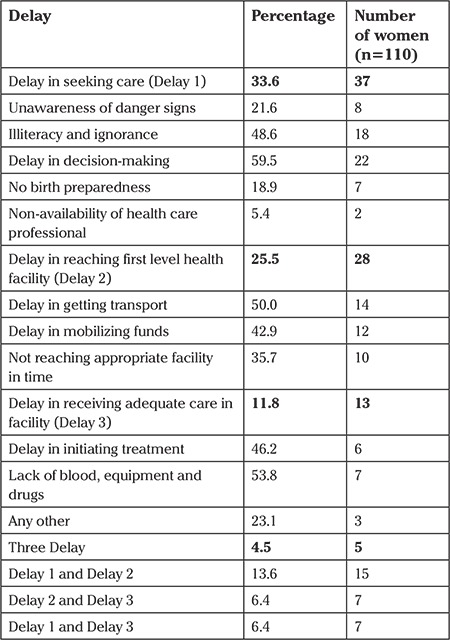
Distribution of deceased women according to delay type

**Table 5:**
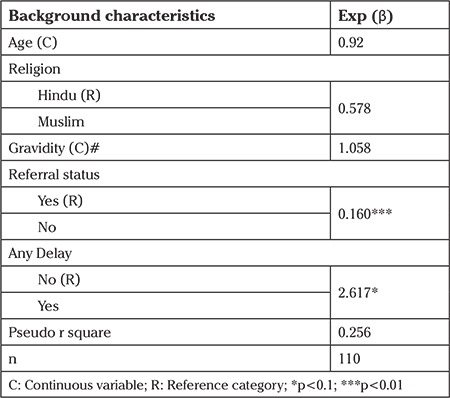
Result of logistic regression showing odds ratio of being unstable at the time of admission

**Table 6:**
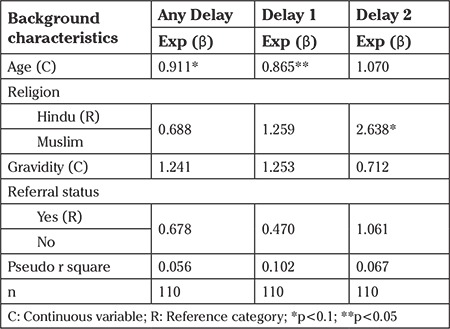
Result of logistic regression showing odds ratio of experiencing any Delay, Delay 1 and Delay 2
